# Prevalence of Psychological Disorders in Patients with Alopecia Areata in Comparison with Normal Subjects

**DOI:** 10.1155/2014/304370

**Published:** 2014-03-09

**Authors:** Shahin Aghaei, Nasrin Saki, Ehsan Daneshmand, Bahare Kardeh

**Affiliations:** ^1^Dermatology Department, Molecular Dermatology Research Center, Shiraz University of Medical Sciences, Shiraz 7134844119, Iran; ^2^Student Research Committee, Shiraz University of Medical Sciences, Shiraz 7134844119, Iran

## Abstract

Alopecia areata is a chronic disease with a great impact on the patient's quality of life. In this study we reviewed the frequency of psychological disorders in patients with alopecia areata in comparison to a control group. We enrolled 40 patients with alopecia areata and a 40-volunteer random age-sex matched control group. The study is based on anxiety and Beck Depression Inventory (BDI) and the Eysenck Personality Questionnaire (EPQ). Analytical evaluation was done by Mann-Whitney, Kruskal Wallis, and *t*-tests. There was a significant difference between the case and control group regarding the prevalence of depression (*P* value = 0.008), anxiety (*P* value = 0.003), and neuroticism (*P* value = 0.05). There was no significant differences regarding extraversion (*P* value = 0.249), psychosis (*P* value = 0.147), and lying (*P* value = 0.899) between the two groups. In alopecia areata involving the head, there was a significant relation only between neuroticism (*P* value = 0.045) and lying (*P* value = 0.005). The facial involvement had a significant relation with depression (*P* value = 0.020), anxiety (*P* value = 0.019), and neuroticism (*P* value = 0.029). The frequency of psychological disorders in the case group is significantly greater than the control group.

## 1. Introduction

Psychosomatic (psychophysiological) medicine has been considered as a particular field of psychology and psychiatry for over 50 years. The history of this branch of medicine is very closely related with the theory of unity of body and mind. Disorders of mind and body and how these two parts of human beings function together is reflected in “The Diagnostic and Statistical Manual of Mental Disorders (DSM),” a criterion for classification of mental disorders [1].

Skin-related mental disorders include a wide range of dermal pathologies which present with psychological signs or stresses. In spite of the evidence on the interactions between nervous, immune, and endocrine systems, which have made the understanding of psychocutaneous diseases easier, further investigations are still required [2]. Skin, as a tangible and visible part of the body, can have a magnificent effect on psychological status which is continuously involved in socialization processes from childhood to adulthood [3, 4].

Alopecia areata is a common chronic disease of skin with sudden onset loss of hair in a clear circular area [5]. The role of psychological factors in extension of alopecia areata has also been discussed. Social and familial problems and uncontrollable events have more influences on these patients than on normal society [6] and most of them experience psychological problems in long-term such as depression, anxiety, and paranoid disorders [6–10]. Also, studies have shown that the low quality of life in these patients has significant relation with depression [11]. It seems that the patients with alopecia areata are mainly depressed, worried, and hysteric, present with higher rates of hypochondriasis tendency, and experience frequent conflicts in daily interactions with other people [12]. Tendency to suicide is high in these patients [13]. Studies have shown that there is a significant relation between loss of hair and stress, stress intensity, and stressful events [14]. There is evidence that besides the medical therapies, hypnotherapy is also effective in treatment of alopecia areata [15, 16].

The purpose of this research is to assess the frequency of psychological disorders in patients with Alopecia areata in comparison with the normal subjects.

## 2. Materials and Method

This case-control study was conducted on patients with Alopecia areata, who had referred to Dermatology Clinic of Jahrom University of Medical Sciences within a one year period. A total of 40 patients were included in our study using simple randomization according to the order they visited the dermatology clinic. They included 44.8% male and 56.2% female. An age-sex matched control group from healthy volunteers who had no history of medical or psychological problems was randomly selected. The study protocol was approved by the Ethics Committee of Shiraz University of Medical Sciences. Informed consent forms were filled by all the patients.

Diagnosis of Alopecia Areata was confirmed by a dermatologist clinically (Shahin Aghaei). Psychological tests included the following: (1) Beck Depression Inventory (1961) which consisted of 21 questions and evaluated the symptoms, physical problems, cognitive, and emotional depression; (2) Beck Anxiety Inventory (1988), which had 21 questions and evaluated physical symptoms, cognitive, and emotional anxiety. (3)* Eysenck Personality Questionnaire* (EPQ) which had 90 items and evaluated four dimensions of extraversion, neuroticism, psychotics, and lying [17]. The obtained results were statistically analyzed by *t*-test, Kruskal Wallis test, and Mann-Whitney test.

## 3. Results

Our patients included 44.8% males and 56.2% females. Personal and family histories of atopy, diabetes mellitus, thyroid diseases, and family histories of alopecia areata were included in Table 1. Frequencies of different locational patterns of alopecia areata were simplified in Table 2.

**Figure 1:**
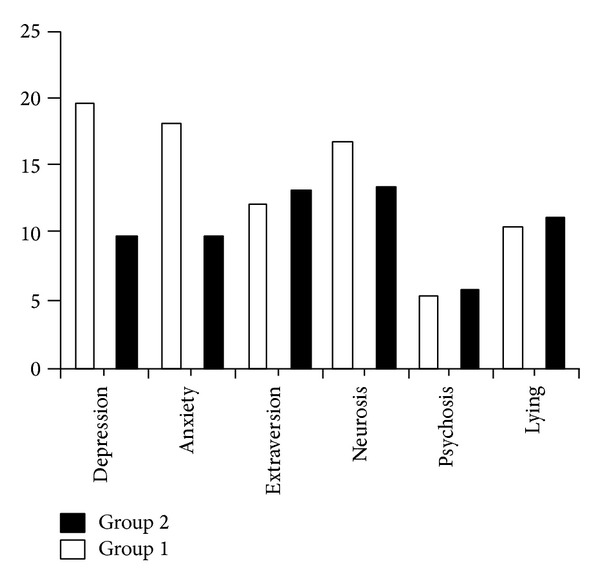
Comparison of the two groups regarding number of cases with depression, anxiety, extraversion, neurosis, psychosis, and lying. (Group  1: case; Group  2: control).

Our study showed that there were significant differences between the case and control groups regarding depression (*P*  value = 0.008), anxiety (*P*  value = 0.003), and neuroticism (*P*  value = 0.05), but no significant differences were detected considering extraversion (*P*  value = 0.249), psychosis (*P*  value = 0.147), and lying (*P*  value = 0.899) (Figure 1).

There was a significant difference between the two groups from the viewpoint of educational level (*P*  value = 0.001), revealing that case group had a lower level of education (Table 3).

There was no significant correlation between duration of the disease, age of onset, number of relapses, and intensity of the disease with anxiety, extraversion, neurosis, psychosis, or lying (*P*  values > 0.05).

In alopecia areata involving the scalp, there were significantly higher rates of neuroticism (*P*  value = 0.045) and lying (*P*  value = 0.005). In the facial involvement, there was significant higher rates of depression (*P*  value = 0.020), anxiety (*P*  value = 0.019), and neurosis (*P*  value = 0.029). There were no significant higher rates of psychological diseases in patients with only eyebrow involvements (*P*  value > 0.05).

## 4. Discussion

Our findings revealed that the rate of depression, stress, and neurosis was higher in the patient group than in the control group, which supports some of the previous researches [2, 3]. This can be explained as follows.

The correlation between alopecia areata and psychological disorders is mutual; on one side the mentioned psychiatric disorders can be considered as a base for initiation and exacerbation of the disease [17, 18] and on the other hand, the disease, through its negative impacts on the patient's life, causes psychological problems [19].

The results show that the patient group had a lower educational level. As previous studies had not concerned evaluation of social backgrounds regarding the item of education, no supportive or contrary results were found. The fact that lower educational level is more prevalent in patients can be best explained as follows. Low education level consequently leads to personal and social problems and vice versa. Problems, which hinder a person from continuing his/her education, can very possibly have undesired impacts on his/her psychological and mental aspects as well. In other words, loss of confidence due to experienced rejection in society makes one vulnerable to more anxiety and stress in confronting difficult conditions. Many studies have confirmed social support as a protective factor for mental and physical health of persons [19].

There was no significant correlation between duration of the disease, age of onset, number of relapses, and intensity of the disease with anxiety, extraversion, neurosis, psychosis, or lying.

It seems that increases in the illness duration, early age of first exposure, and frequent episodes decrease the rate of depression and anxiety. It means the patients experienced more anxiety and depression in the first episode which attenuates over time and with repeated periods, although it does not disappear completely. This can be logically explained by chronicity of the illness and experience of similar conditions. On the other hand, the percent of hope to recovery from the patient's view has inverse relation with increase in depression severity, anxiety, and neurosis, although no significant relation was detected in this regard. This fact may originate from the patient's negative attitude toward himself, which leads to lack of self-confidence, disappointment, lack of pleasure and satisfaction in life, and denial of the disease by the patient.

The results showed that in alopecia areata involving the scalp, there were significant higher rates of neurosis and lying. If there were any facial involvements, there were significant rates of depression, anxiety, and neurosis. These results are consistent with previous results [20]. The most important variable that leads to mental illnesses in skin disorders is deformation [20], which means, regarding to the size and place of the lesions, the psychological reactions can be very different. [21].

## 5. Conclusion

Generally, it can be conceived that the prevalence of mental disorders in the case group is higher than the control group; hence, a proper awareness and consciousness about the psychosocial and professional backgrounds is essential for desirable management of the disease.

## Figures and Tables

**Table 1:** Personal and family history of patients.

Personal and family history	Number of patients	% of patients
Personal history of atopy	8	20
Family history of atopy	7	17.5
Personal history of diabetes mellitus	1	2.5
Family history of diabetes mellitus	8	20
Personal history of thyroid disease	1	2.5
Family history of thyroid disease	4	10
History of alopecia in first-degree relatives	3	7.5

**Table 2:** Frequency of various patterns of invovlement.

Body involvement	Nail involvement	Eyelash involvement	Eyebrow involvement	Face involvement	Scalp involvement
2	2	1	4	10	31

**Table 3:** Educational level in patients and controls.

Educational Level	Patient group	Control group
College	37.5%	75%
Diploma	32.5%	20%
Lower than Diploma	30%	5%
Total	**100%**	**100%**
